# Community Outreach for Navajo People Living with Diabetes: Who Benefits Most?

**DOI:** 10.5888/pcd17.200068

**Published:** 2020-07-23

**Authors:** Letizia Trevisi, John E. Orav, Sidney Atwood, Christian Brown, Cameron Curley, Caroline King, Olivia Muskett, Hannah Sehn, Adrianne Katrina Nelson, Mae-Gilene Begay, Sonya S. Shin

**Affiliations:** 1 Department of Global Health and Social Medicine, Harvard Medical School, Boston, Massachusetts; 2 Department of Medicine, Brigham and Women’s Hospital, Boston, Massachusetts; 3 Division of Global Health Equity, Brigham and Women’s Hospital, Boston, Massachusetts; 4 School of Medicine, Oregon Health and Science University, Portland, Oregon; 5 Partners in Health, Boston, Massachusetts; 6 Navajo Nation Department of Health, Navajo Department of Health, Window Rock, Navajo Nation

## Abstract

**Introduction:**

The Community Outreach and Patient Empowerment (COPE) intervention provides integrated outreach through community health representatives (CHRs) to people living with diabetes in Navajo Nation. The aim of this study was to identify groups for whom the intervention had the greatest effect on glycated hemoglobin A_1c_ (HbA_1c_).

**Methods:**

We analyzed de-identified data extracted from routine health records dated from December 1, 2010, through August 31, 2014, to compare net change in HbA_1c_ among COPE patients and non-COPE patients. We used linear mixed models to assess whether the intervention was modified by age, sex, preferred language, having a primary care provider, baseline HbA_1c_, or having a mental health condition.

**Results:**

Age, having a primary care provider, and baseline HbA_1c_ significantly modified HbA_1c_ levels. Among patients aged 64 or younger, COPE participation was associated with a net decrease in HbA_1c_ of 0.77%; among patients aged 65 or older, the net decrease was 0.49% (*P* = .03). COPE participation was associated with a steeper decrease in HbA_1c_ among patients without a primary care physician (net decrease, 0.99%) than among patients with a primary care provider (net decrease, 0.57%) (*P* = .03). COPE patients with a baseline HbA_1c_ >9% had a net decrease of 0.70%, while those with a baseline HbA_1c_ ≤9% had a net decrease of 0.34% (*P* = .01). We found no significant differences based on sex, preferred language, or having a mental health condition.

**Conclusion:**

Findings suggest that the COPE intervention was robust and equitable, benefiting all groups living with diabetes in Navajo Nation, but conferring the greatest benefit on the most vulnerable.

**Summary**

**What is already known on this topic?**

Programs that train and deploy community health representatives (CHRs), community health workers for tribal communities, can improve health outcomes for people with chronic disease.

**What is added by this report?**

Patient groups who benefitted most from a CHR program for people with diabetes in Navajo Nation were aged 64 or younger, did not have a primary care provider, and had a baseline HbA_1c_ >9%. Older patients, patients with a primary care provider, and patients with a baseline HbA_1c_ ≤9% also had significant improvements in glycemic levels.

**What are the implications for public health practice?**

Our findings can inform program delivery by identifying patients’ groups to refer to community-based programs and highlight how to strengthen the CHR program to better meet the needs of less responsive groups.

## Introduction

American Indian and Alaska Native populations in the United States have a disproportionate burden of type 2 diabetes (1). The erosion of traditional lifeways and culturally estranged health care systems — present-day manifestations of colonization — are major drivers of diet-related health disparities (2–4). Conversely, cultural continuity and self-determination (the sovereign right of tribal nations to govern themselves) protect against diabetes risk among Indigenous communities (5). The Community Health Representative (CHR) program is a unique asset in tribal health care systems that embodies both cultural preservation and self-determination. A tribally run program established in 1968, the CHR Program employs “frontline public health workers who are trusted members of the community with a close understanding of the community, language, and traditions” (6). CHRs provide services to people living on the reservation who typically face barriers to health care. They are particularly effective health care professionals because they address both clinical and social factors that contribute to health, providing clinical care and health education and addressing housing, nutritional, and psychosocial needs.

To promote the integration of CHRs in clinic-based health care teams, the Community Outreach and Patient Empowerment (COPE) Project was launched in 2010 to improve patient care for medically underserved people living with chronic conditions, including diabetes. A team of global health providers from Brigham and Women’s Hospital partnered with the Navajo Nation CHR Program and Navajo Area Indian Health Services to conduct this program. The COPE Project sought to enhance the central role of CHRs in their communities and connect them more closely to clinic-based providers. The COPE intervention focused on increasing provider referrals of patients living with diabetes to Navajo CHRs, training CHRs to deliver standardized health promotion in homes, and strengthening care coordination between CHRs and clinic-based providers. Our previous research demonstrated that COPE was associated with significant improvements in glycemic and lipid control 2 years after patient enrollment (7).

Despite numerous studies describing interventions to improve diabetes outcomes among American Indian/Alaska Native populations (8), few studies have systematically sought to identify patient characteristics associated with the effect modification of the intervention. To inform how best to scale up such an intervention, identifying which groups of patients benefit most from the outreach is crucial. For instance, if only certain groups benefit, the intervention could be targeted to those groups, and alternative interventions could be sought to address the needs of other groups. The objective of our study was to identify groups of people who benefit most from the COPE intervention among people living with diabetes in Navajo Nation.

## Methods

### Study setting and health care system

The study was conducted in the Navajo Nation, a reservation with a population of 350,000 (9) that covers more than 27,000 square miles of Arizona, New Mexico, and Utah. The Navajo Area Indian Health Service is one of the 12 regional administrative units of the national Indian Health Service, with an active user population of 247,000 people. The Navajo Department of Health oversees the tribal CHR Program, comprising nearly 100 CHRs who are certified nursing assistants and fluent in Navajo. Currently, an estimated 100,000 Navajo, about half of the adult population, are living with either type 2 diabetes or prediabetes (10), compared with 9.4% of the adult US population (11), 50% in Arizona, 43% in Utah, and 54% in New Mexico (12–14).

### Program intervention

The COPE intervention aims to improve health care delivery by positioning CHRs as valuable and integral members of the health care delivery team. Despite mounting evidence that community health worker programs are most effective when integrated into formal health care systems (15,16), health care delivery systems often lack the tools to create effective community–clinical linkages. The World Health Organization has highlighted the need for replicable best practices that can improve the way in which community health workers are embedded in health care teams through both system and policy change (17). The COPE intervention targets systems-level change to integrate community health workers into health care systems. COPE defines the care team as a triad of clinic-based providers, CHRs, and patients and implements 3 strategies to strengthen and standardize the relationship between each participant group: patient referral to the Navajo CHR Program, standardized delivery of community-based accompaniment, and community–clinical linkages. 

**Patient referral to the Navajo CHR Program**. Clear referrals pathways were established for providers to refer patients to the CHR Program. Criteria for referral and enrollment in COPE were deliberately broad: any person seen at a participating health facility who was deemed to have a clinical indicator (eg, an elevated glycated hemoglobin A_1c_ [HbA_1c_], a comorbid condition such as hypertension or elevated cholesterol, diabetes complications such as neuropathy or retinopathy) or a psychosocial risk factor (eg, minimal social support, geographic isolation, lack of engagement with a health care provider) was referred to the program.

**Standardized delivery of community-based accompaniment**. Community-based accompaniment refers to a model of community health worker support through home visits to provide care and support to patients to address geographic, social, and economic barriers to care. CHRs were trained in evidence-based behavior-change strategies, including motivational interviewing and goal setting, and received structured teaching materials (flipcharts) to deliver health promotion information to their clients. Each flipchart used culturally informed language and imagery and was organized according to a brief counseling sequence known as the 5A’s (Ask, Assess, Advise, Assist, Arrange follow-up) (18,19). At home visits, CHRs typically checked vital signs, delivered health coaching, and facilitated goal setting. 

**Community–clinical linkages.** Community–clinical linkages are defined by the Centers for Disease Control and Prevention as connections between community and clinical sectors to improve population health (20). CHRs and providers established systems to coordinate patient care, including electronic referrals and CHR access to electronic health records. The intervention is described elsewhere (7).

### Study design

We abstracted de-identified data from electronic health records dated December 1, 2010, through August 31, 2014, of adults aged 18 or older who had an *International Classification of Disease, 10th Revision, Clinical Modification* (ICD-10) diagnosis code for type 2 diabetes and were receiving care at a participating health care facility. We abstracted sociodemographic and clinical data from the Resource and Patient Management System, the electronic health record used by most Indian Health Service facilities for routine clinical care. We identified COPE participants in the database and matched them to non-COPE participants based on age (±5 y), sex, primary health facility, HbA_1c_ (±1 point), and systolic blood pressure (±10 mm Hg) at baseline (ie, in the 3 months before the date of COPE enrollment). For matched non-COPE patients, we assigned the enrollment date of their matched COPE patient. This study was approved by the Partners Healthcare Institutional Review Board and the Navajo Nation Human Research Review Board. The data extraction process took place from December 1, 2013, through August 31, 2016.

### Study outcome and potential effect modifiers

The primary outcome was the change in HbA_1c_ from baseline to 2 years after enrollment. We evaluated whether these changes among COPE and non-COPE patients were moderated by patient characteristics. We selected baseline characteristics on the basis of a literature review and input from Navajo stakeholders, including a community health advisory panel, a committee of patients, family members, and CHRs, who provided advisory input throughout the study. We tested the following binary variables for effect modification: age (>median or ≤median), sex (male or female), preferred language (English or Indigenous language), having a primary care provider (PCP) at enrollment (yes or no), ICD-10 diagnosis of depression (yes or no), ICD-10 diagnosis of alcohol and/or drug dependence (yes or no), and baseline HbA_1c_ (>9% or ≤9%). We chose an HbA_1c_ value of >9% because this value is often used as a clinical threshold to indicate people with poor glycemic control and increased risk of diabetes complications (21).

### Data analysis

We evaluated differences in baseline characteristics between COPE and non-COPE patients by using χ^2^ tests. We conducted mixed linear regression models to assess whether differences over time in HbA_1c_ among the 2 groups were altered by potential effect modifiers. We assessed each potential effect modifier in a separate model. To ensure comparability between the intervention and nonintervention group, we adjusted for baseline HbA_1c_ as averaged over the 2 years before the enrollment date. We accounted for within-patient and within-facility correlation with a random effect for each patient and for each primary health facility. The primary predictors of the model were time, a binary variable that indicates whether the outcome is measured before enrollment or after enrollment; COPE, a binary variable that differentiates between COPE and non-COPE patients; factor, the potential effect modifier of interest; and all interaction terms. In the sensitivity analyses, we also adjusted the models for each matching set of COPE and non-COPE patients. For the multivariate models, we included covariates identified a priori as potential confounders: age, sex, preferred language, having a PCP, and ICD-10 diagnoses of essential hypertension (a diagnosis of hypertension with no identifiable cause), major depression disorder, substance use disorder, dyslipidemia, and major cardiovascular disease. Major cardiovascular disease was defined as having at least 1 of the following diagnoses: acute myocardial infarction, coronary artery bypass surgery, coronary angioplasty, peripheral arterial disease, abdominal aortic aneurysm, carotid artery disease, or cerebrovascular disease. To verify our primary analyses, we conducted stratified analyses by each potential modifier. We conducted all analyses in January 2020 by using SAS version 9.4 (SAS Institute, Inc), and a 2-sided *P* value <.05 was considered significant.

## Results

We identified 173 COPE patients and 2,880 non-COPE patients who had type 2 diabetes and received care at a participating health care facility. Most (approximately 77%) patients in both groups were aged 56 or older, and most were female (62.4% among COPE patients and 68.7% among non-COPE patients) (Table 1). Diagnoses of essential hypertension (65.3% among COPE patients and 68.3% among non-COPE patients) and major cardiovascular disease (71.1% among COPE patients and 71.2% among non-COPE patients) were prevalent. Compared with non-COPE patients, COPE participants were less likely to report English as their preferred language (41.6% vs 59.0%, *P* < .001), more likely to have a substance use disorder (7.5% vs 3.4%, *P* = .005), less likely to have dyslipidemia (46.2% vs 58.6%, *P* = .001), and more likely to have a baseline HbA_1c_ >9% (47.4% vs 22.3%, *P* < .001).

**Table 1:** Baseline Characteristics of COPE and Non-COPE Participants, Navajo Nation, United States, 2010–2014

Characteristic	COPE, No. (%) (n = 173)	Non-COPE, No. (%) (n = 2,880)	*P* Value^a^
**Sociodemographic**			
**Age, y**			
25–40	7 (4.0)	46 (1.6)	.003
41–55	33 (19.1)	610 (21.2)	
56–70	74 (42.8)	1,436 (49.9)	
71–85	54 (31.2)	764 (26.5)	
≥86	5 (2.9)	24 (0.8)	
**Sex**			
Male	65 (37.6)	901 (31.3)	.08
Female	108 (62.4)	1,979 (68.7)	
**Preferred language**			
Indigenous	100 (57.8)	1,178 (40.9)	<.001
English	72 (41.6)	1,700 (59.0)	
Missing	1 (0.6)	2 (0.1)	
**Has a primary care provider at enrollment**			
Yes	146 (84.4)	2,577 (89.5)	.04
No	27 (15.6)	303 (10.5)	
**Diagnoses**			
**Essential hypertension^b^**			
Yes	113 (65.3)	1,966 (68.3)	.40
No	60 (34.7)	909 (31.6)	
Missing	0	5 (0.2)	
**Major depression disorder**			
Yes	25 (14.4)	282 (9.8)	.05
No	148 (85.6)	2,593 (90.0)	
Missing	0 (0)	5 (0.2)	
**Substance use disorder**			
Yes	13 (7.5)	97 (3.4)	.005
No	160 (92.5)	2,778 (96.5)	
Missing	0	5 (0.2)	
**Major cardiovascular disease^c^**			
Yes	123 (71.1)	2,050 (71.2)	.95
No	50 (28.9)	825 (28.6)	
Missing	0	5 (0.2)	
**Dyslipidemia**			
Yes	80 (46.2)	1,688 (58.6)	.001
No	93 (53.8)	1187 (41.2)	
Missing	0	5 (0.2)	
**HbA_1c_ at baseline^d^**			
≤9%	91 (52.6)	2,239 (77.7)	<.001
>9%	82 (47.4)	641 (22.3)	

Abbreviations: COPE, Community Outreach and Patient Empowerment; HbA_1c_, glycated hemoglobin A_1c_.

a χ^2^ test or Fisher exact test.

b Essential hypertension is the form of hypertension that has no identifiable cause.

c Defined as ≥1 of the following diagnoses: acute myocardial infarction, coronary artery bypass surgery, coronary angioplasty, peripheral arterial disease, abdominal aortic aneurysm, carotid artery disease, cerebrovascular disease.

d Evaluation at the closest available HbA_1c_ measure before the enrollment date.

In the adjusted analyses, the association between the intervention and HbA_1c_ values over time was significantly modified by the following baseline factors: age, having a PCP, and baseline HbA_1c_ (Table 2). We found no significant changes based on sex, preferred language, depression disorder, or substance use disorder. For significant effect modifiers (age, having a PCP, and baseline HbA_1c_), COPE participation was associated with significant HbA_1c_ improvements in all 6 groups (aged ≤64 and ≥65, having a PCP and not having a PCP, HbA_1c_ >9% and HbA_1c_ ≤9%) (Table 2). However, the net change in HbA_1c_ associated with COPE participation was greater among patients aged 64 or younger, patients without a PCP, and patients with a baseline HbA_1c_ >9%. We detected a net decrease in HbA_1c_ of 0.77% (95% confidence interval [CI], 0.59%–0.95%) among patients aged 64 or younger and a net decrease in HbA_1c_ among patients aged 65 or older of 0.49% (95% CI, 0.31%–0.67%). HbA_1c_ levels declined more steeply in the COPE patients without a PCP (0.99%; 95% CI, 0.64%–1.35%) than among COPE patients with a PCP (0.57%; 95% CI, 0.44%–0.71%). Finally, COPE patients with a baseline HbA_1c_ >9% had a larger net decrease in HbA_1c_ (0.70% [95% CI, 0.51%–0.88%]) than COPE patients with a baseline HbA_1c_ ≤9% (0.34% [95% CI, 0.16%–0.52%]). The groups with the greatest relative benefit from COPE were patients without a PCP (compared with patients with a PCP) and patients with a baseline HbA_1c_ >9% (compared with a baseline HbA_1c_ ≤9%).

**Table 2:** Intraclass Differences in HbA_1c_ Least Squares Means at Baseline and 2 Years After Enrollment in COPE Intervention and Difference in Differences in the Adjusted Model^a^, Navajo Nation, United States, 2010–2014

Class	COPE vs Non-COPE	Difference Between Baseline and 2 Years After Enrollment (95%CI)	DID (95% CI)	*P* Value^b^
**Age, y**				
≤64	Non-COPE	0.09 (0.05 to 0.14)	0.77 (0.59 to 0.95)	.03
	COPE	−0.68 (−0.85 to −0.50)		
≥65	Non-COPE	0.03 (−0.01 to 0.08)	0.49 (0.31 to 0.67)	
	COPE	−0.46 (−0.63 to −0.28)		
**Sex**				
Female	Non-COPE	0.10 (0.06 to 0.14)	0.62 (0.46 to 0.78)	.99
	COPE	−0.53 (−0.68 to −0.37)		
Male	Non-COPE	0.01 (−0.05 to 0.06)	0.63 (0.41 to 0.84)	
	COPE	−0.62 (−0.82 to −0.42)		
**Language preference**				
English	Non-COPE	0.08 (0.04 to 0.12)	0.74 (0.56 to 0.93)	.11
	COPE	−0.66 (−0.85 to −0.48)		
Indigenous	Non-COPE	0.06 (0.01 to 0.10)	0.53 (0.36 to 0.71)	
	COPE	−0.48 (−0.64 to −0.31)		
**Has a primary care provider at enrollment**				
No	Non-COPE	−0.01 (−0.12 to 0.09)	0.99 (0.64 to 1.35)	.03
	COPE	−1.01 (−1.34 to −0.67)		
Yes	Non-COPE	0.08 (0.05 to 0.11)	0.57 (0.44 to 0.71)	
	COPE	−0.49 (−0.63 to −0.36)		
**Has major depression disorder**				
Yes	Non-COPE	0.14 (0.04 to 0.23)	0.91 (0.60 to 1.22)	.06
	COPE	−0.77 (−1.07 to −0.48)		
No	Non-COPE	0.06 (0.03 to 0.10)	0.58 (0.44 to 0.72)	
	COPE	−0.51 (−0.65 to −0.38)		
**Substance use disorder**				
Yes	Non-COPE	0.18 (0 to 0.36)	0.25 (−0.22 to 0.73)	.10
	COPE	−0.07 (−0.51 to 0.37)		
No	Non-COPE	0.07 (0.04 to 0.10)	0.67 (0.54 to 0.80)	
	COPE	−0.60 (−0.73 to −0.47)		
**HbA_1c_ at baseline**				
≤9%	Non-COPE	0.15 (0.12 to 0.19)	0.34 (0.16 to 0.52)	.01
	COPE	−0.19 (−0.37 to −0.01)		
>9%	Non-COPE	−0.21 (−0.28 to −0.15)	0.70 (0.51 to 0.88)	
	COPE	−0.91 (−1.08 to −0.74)		

Abbreviations: HbA_1c_, glycated hemoglobin A_1c_; DID, difference in differences; CI, confidence interval; COPE, Community Outreach and Patient Empowerment.

a Adjusted model for pre-2 years’ means, age (years, continuous), sex (male/female), preferred language (English/Indigenous), primary care provider (yes/no); essential hypertension (yes/no), major depression disorder (yes/no), substance use disorder (yes/no), dyslipidemia (yes/no), and major cardiovascular disease (yes/no).

b Derived from linear mixed models.

## Discussion

In our study, we sought to understand which patients benefited most from a CHR-delivered diabetes outreach intervention in Navajo Nation. We compared the net change in HbA_1c_ at 2 years from baseline to postenrollment among COPE participants and nonparticipants and tested whether any sociodemographic or clinical baseline characteristics predicted a greater relative response to the intervention. COPE participation resulted in improved glycemic levels overall. Our findings identified several groups that benefited most from the COPE intervention, namely participants aged 64 or younger, participants without a PCP at enrollment, and participants whose baseline HbA_1c_ was >9%.

Patients without a PCP benefited more from the intervention than patients who had already been assigned to a PCP. The integration of community health workers into primary care teams has been effective and cost-effective in diabetes care (22,23); however, to our knowledge, ours is the first study to demonstrate that patients who lack a PCP, compared with patients who have a PCP, may benefit even more from community health worker outreach. We speculate that such patients were receiving less primary care before enrolling in COPE and subsequently began to engage more in health care services with CHR support. Through CHR encouragement and support, patients may be better equipped to navigate the health care system, which can otherwise be overwhelming, especially for people without a PCP. For instance, one flipchart, “Getting Care from Clinic,” coaches patients on how to best communicate with providers and advocate for patient-centered care. Thus, patients who are least engaged in primary care services at baseline may be most likely to benefit from the intervention. This finding is particularly important because it suggests that CHR outreach may be an effective strategy for a particularly vulnerable group — people living with diabetes who are not yet engaged in primary care.

We also found that patients with a baseline HbA_1c_ >9% had a larger intervention effect than patients with a baseline HbA_1c_ ≤9%. These findings are consistent with several systematic reviews of health care interventions among people living with type 2 diabetes, which also found that people with high baseline HbA_1c_ values had greater improvements in follow-up (24,25). This observation is not surprising, because a patient beginning with an HbA_1c_ >9% will require greater absolute changes in HbA_1c_ values to achieve target ranges, in comparison to a patient beginning with an HbA_1c_ ≤9%. Furthermore, patients who begin with a high HbA_1c_ may be more motivated to make changes because they either have physical symptoms of hyperglycemia or understand their risk of future complications to be greater.

The finding that patients aged 64 or younger had a greater response to the intervention than patients aged 65 or older is particularly salient, in light of the fact that type 2 diabetes is affecting Navajo and other Native populations at increasingly younger ages (26). A systematic review of tailored integrated diabetes primary care interventions found the opposite, namely that younger people, compared with older people, had higher HbA_1c_ values in follow-up (25). Outreach that is effective at engaging younger people and achieving sustained improvements in glycemic levels could contribute substantially to reducing the burden of complications as these younger people age.

COPE was still effective among older patients, although to a lesser degree. Patients aged 65 or older might depend on caretakers to purchase and prepare food and may have greater limitations in physical activity. Our qualitative research (27) showed that older patients made more lifestyle changes when CHRs actively engaged relatives and caregivers during home visits and encouraged family members to be involved in the patient’s health and well-being. An assessment of older patients’ independence and, if relevant, more structured CHR-delivered support to caregivers could further enhance treatment response for older people.

Our study adds to the scarce literature that supports culturally informed interventions to improve diabetes outcomes in Native communities. To our knowledge, our study findings generate unique evidence to support multilevel interventions in Native communities by demonstrating benefits on clinical outcomes (7) and highlighting groups who may benefit the most from the intervention. People aged 64 or younger, people with an HbA_1c_ ≤9%, and people without a PCP represent groups who stand to benefit greatly from improved glycemic control (25). These results can inform program delivery by identifying which patients to refer to community-based programs, in particular, CHR outreach. Furthermore, the findings highlight opportunities to further strengthen the model to better address the needs of less responsive groups. Less responsive groups (eg, patients aged ≥65, patients with a PCP, patients with HbA_1c_ ≤9%) did have significant improvements associated with COPE participation, although to a lesser degree than their counterparts. Nonetheless, our findings will be used to enhance the program to better meet the needs of older patients.

Our study has several limitations. First, we relied on medical record data collected as part of routine care. Laboratory values and ICD-10 diagnoses may be missing or underreported because of underuse of health care services as a result of various factors, such as vast geographic distances to access health care services, lack of public transportation, lack of insurance, and substantial turnover of health care providers (common in medically underserved areas affected by health disparities). Second, this study was limited to people living with type 2 diabetes who were seen at a participating facility. Thus, findings may not be representative of patients living with diabetes who were not engaged in health care at all or were seen at other facilities. We could have underestimated the effect of our intervention if the positive effects of the intervention spilled over to non-COPE patients at participating sites, because the CHRs who saw COPE patients also saw non-COPE patients. However, we estimate that the non-COPE patients seen by CHRs would represent a small proportion of the entire nonintervention group (<5%). Finally, long-standing CHR programs exist across American Indian/Alaska Native communities in the United States and Canada, and the scope of work performed by CHRs is heterogeneous across programs. The findings of any study on CHR interventions among American Indian/Alaska Native populations must be considered in the context of each tribal CHR program and in the broader context of multilevel community health worker interventions.

On the other hand, we consider COPE’s programmatic implementation to be a unique strength of this study. As a nonresearch, “real-world” program, our findings reflect patients who were referred to an existing program without the constraining aspects of research participation, such as informed consent, remuneration, and data collection. In this sense, our observational research of a local standard-of-care service may have greater generalizability to inform future program implementation. Future research may include examining the effect of the COPE intervention among people living with diverse chronic diseases and among people in other communities, such as other tribal populations and other minority populations.

The COPE intervention resulted in significant improvements in glycemic control among all groups, suggesting a robust benefit among Navajo people living with diabetes. Nonetheless, the intervention had the greatest effect among 3 groups: patients aged 64 or younger, patients without a PCP at enrollment, and patients with a baseline HbA_1c_ >9%. These findings suggest the program was successful in advancing health equity, namely conferring the greatest benefit to the most vulnerable groups. Programmatic efforts to integrate CHRs in tribal health care systems have the potential to advance Native health equity.
